# Historical changes in baby names in China

**DOI:** 10.12688/f1000research.131990.1

**Published:** 2023-06-05

**Authors:** Yuji Ogihara

**Affiliations:** 1 Tokyo University of Science, Tokyo, Japan

**Keywords:** name, uniqueness, historical change, cultural change, individualism, China, need for uniqueness, culture

## Abstract

Based on previous research on names and naming practices, I propose three suggestions to Bao et al. (2021), which investigated historical changes in given names of Han Chinese in China between 1920 and 2005. Their study analyzed a one-shot cross-sectional survey conducted in 2005 and reported that unique names increased from 1920 to 2005. The authors concluded that China became more individualistic over time for the period. However, three questions have remained unanswered in Bao et al. (2021). First, were the samples of older birth cohorts truly representative? Second, did unique names increase only after the 1970s? Third, how are the historical changes in average name length interpreted? Answering these three questions would contribute to a further understanding of the historical changes in given names and their underlying psychological/cultural shifts in China.

I have conducted research on unique names (e.g., Ogihara, 2015, 2021a, 2021b, 2021c, 2023b; Ogihara *et al*., 2015; Ogihara & Ito, 2022) and related cultural changes (e.g., Ogihara *et al*., 2016; Ogihara, 2018b, 2023a; for reviews, see Ogihara, 2017, 2018a). Based on this previous research, I suggested three recommendations ( Ogihara, 2020) to a prior study on historical changes in baby names in China (Study 2 in Cai *et al*., 2018). The authors answered some of my comments with an empirical investigation on a new dataset ( Bao *et al*., 2021). I respond to it by focusing on three major points.

Specifically, Bao *et al*. (2021) examined historical changes in given names of Han Chinese in China between 1920 and 2005 by analyzing a cross-sectional survey. This article provides rich information about historical changes in names in China and their underlying psychological/cultural shifts.

## 1. Were the samples of older birth cohorts truly representative?

The authors used a random subset of a one-shot cross-sectional survey conducted in 2005 (the 2005 China’s 1% Population Census) and analyzed given names of people born between 1920 and 2005. They emphasized that the sample is representative (e.g., “Using a large representative sample of Chinese names” in Abstract, “We used an unprecedentedly large representative sample of Chinese names, covering a longer period of time from 1920 to 2005” (p. 4) in Discussion, “To obtain a nationally representative sample of Chinese names covering a long period” (p. 2) in Method).

However, the data is from a one-shot cross-sectional survey, not a cross-temporal survey (e.g., birth records). The authors investigated names of Chinese people aged from 0 (newborns) to 85 years. This indicates a possibility that the samples for some populations, especially older birth cohorts, may not be nationally representative (not including all the names given in a year in China). Considering that the average life expectancy in China in 2005 was approximately 73 years (72.99; United Nations, 2022), especially the data for older people would be systematically selected by death, yielding the selection effect. For example, economically wealthy people would live longer (despite diseases and aging, e.g., Wilkinson & Marmot, 2003; Jagger *et al.*, 2008), and physically healthy people would be better suited to survive natural disasters at a higher rate, leading to the possibility that economically not wealthy and physically not healthy older people were underrepresented in the samples. In other words, although a subset of the 2005 China’s 1% Population Census would represent people who lived in 2005, older birth cohorts would not be representative, implying that the results for older years might not reflect the reality. To avoid these systematic biases, previous research examining historical changes in baby names analyzed cross-temporal data. Prior research in China (e.g., Cai *et al*., 2018), Japan (e.g., Ogihara, 2021a, 2022a; Ogihara *et al*., 2015; Ogihara & Ito, 2022), the United States (e.g., Ogihara, 2021d; Twenge *et al*., 2010, 2016), the United Kingdom (e.g., Bush, 2020; Bush *et al*., 2018), Germany (e.g., Gerhards & Hackenbroch, 2000), and France (e.g., Mignot, 2022) has used a series of yearly cross-temporal data of newborn baby names.

It would be necessary for the authors to clarify how they overcame these possible biases. The authors already stated that “because the sample sizes for birth years < 1920 were not sufficient, we limited the range of birth years to 1920~2005” (p. 2), but this issue is related to not only sample size but also sample characteristics (selection bias). The sample sizes for the earlier periods between 1920 and 2005 would not be sufficient to claim that the samples are representative, and the samples would be systematically selected and biased. Cross-sectional data should be carefully investigated to discuss cross-temporal changes (e.g., Cai *et al*., 2018; Ogihara, 2022b; Ogihara & Kusumi, 2020; Twenge, 2011; Twenge & Campbell, 2001). ^1^

## 2. Did unique names increase only after the 1970s?

The authors concluded that unique names increased in China between 1920 and 2005 and claimed that they replicated their previous study, which insists on an increase in unique names between 1950 and 2009 ( Cai *et al*., 2018).

However, all six indicators the authors analyzed consistently showed that the unique names did not increase from 1920 to 1969. Rather, the indicator of name-character uniqueness, which the authors “preferred” (p. 6) most and stated “the estimation would be more accurate” (p. 6) among all six indicators, shows a gradual decrease in uniqueness from 1920 to 1969 (Figure 2B in Bao *et al*., 2021). These results were inconsistent with their previous finding that insists on a continuous increase in unique names from 1950 to 2009 ( Cai *et al*., 2018). The authors did not mention this point clearly. ^2^ The study would be improved if the authors made efforts to explain why unique names did not increase between 1920 and 1969 and why the study did not replicate the previous finding.

One possible reason is the above-mentioned plausible biases in the samples. As I explained above, the samples in the older birth cohorts would likely include a higher proportion of more economically wealthy people. Previous research has demonstrated that people of high economic status tend to express more uniqueness (e.g., Ma *et al*., 2017; Snibbe & Markus, 2005; Stephens *et al*., 2007; Wang *et al*., 2020). Thus, the values of the uniqueness indicators in the older birth cohorts would be higher than the actual values and should be lower in reality. If this is true, an increase in unique names would be observed from 1920 to 1969 as well as from 1970 to 2005, showing that unique names would continue to increase from 1920 to 2005.

## 3. How are the historical changes in average name length interpreted?

The historical changes in average name length (described in Figure 2F in Bao *et al*., 2021) were newly added to a previous study ( Cai *et al*., 2018). They showed a different pattern of changes from those of character-based indices and seem to be divided into three periods: 1) 1920-1960: almost stable (maintained), 2) 1961-1990: sharp decrease, and 3) 1991-2005: sharp increase ( Table 1). ^3^

**Table 1.** Historical changes in average name length of given names and proportions of one-character and three-character given names in China ( Bao *et al*., 2021).

	Average name length	Proportion of one-character names	Proportion of three-character names
1920-1960	Stable	Stable	Stable
1961-1990	Decrease	Increase	Stable
1991-2005	Increase	Decrease	Increase

Note. This table is based on Bao *et al.* (2021)’s Figure 2.

However, the authors did not explain these changes and possible interpretations sufficiently. These drastic changes might be related to various changes in official rules regarding names, political policies, and so on (e.g., Ogihara, 2020). These changes in social, economic, and political aspects should also be considered when cultural changes are discussed.

The analysis shows that given names of Han Chinese in China typically consisted of two Chinese characters at least between 1920 and 2005 (Figure 1 in Bao *et al*., 2021). From 1920 to 1960, the proportions of one-character and three-character names did not change extensively, leading to the stability of the average name length. From 1961 to 1990, the proportion of one-character names remarkably increased (from approximately 10% to over 30%), but the proportion of three-character names did not vary, which decreased the average name length. It would be beneficial to investigate why only the proportion of one-character names remarkably increased during this period. From 1991 to 2005, the proportion of three-character names increased and the proportion of one-character names decreased, causing the increase in the average name length of this period. It would also be important to examine why the proportion of three-character names increased but the proportion of one-character names decreased.

## Conclusion

I propose three suggestions that would further increase the validity and impact of the article ( Bao *et al*., 2021). First, it would be better to answer whether the samples of older birth cohorts were truly representative. Second, it would be preferrable to answer whether unique names increased only after the 1970s. Third, it should be clarified how the historical changes in average name length are interpreted. These suggestions would hopefully contribute to a further understanding of the historical changes in baby names and their underlying psychological/cultural shifts in China.

## Ethics statement

Not applicable.

## Data Availability

No data is associated with this article.

## Footnotes

^1^

Furthermore, because some people changed their given names, their names in 2005 are different from the names given at births. This possibility should also be considered when cross-sectional data are used to examine cross-temporal changes in names.

^2^

Cai *et al*. (2018) also showed this possibility (unique names increased only after 1970). The average name character frequency per birth cohort of 1970-1979 was higher than that of 1960-1969 (Figure 2 in Cai *et al*., 2018). However, this might be solely due to small sample sizes ( Ogihara, 2020).

^3^
However, the possible bias in the samples, which I discuss before, should be considered here, too.
