# Supplementary material for: Historical changes in baby names in China
Source: F1000Res. 2023 Nov 8;12:601. Originally published 2023 Jun 5. [Version 2] doi: 10.12688/f1000research.131990.2 (PMC10840086; doi:10.12688/f1000research.131990.2)
Supplement: Peer review history

# Reviewer report: Reviewer response for version 2

**DOI:** 10.5256/f1000research.158443.r221652

**Published:** 2024-01-10

**Authors:** Shintaro Fukushima

**Affiliations:** 1 Tokyo Woman's Christian University, Tokyo, Japan

Thank you for your reply. I requested to clarify the fact that unique names, but not uniqueness, of the participants truly results in low life expectancy. In response to this point, the author added the two points; 1) people of high economic status tend to express more uniqueness, and 2) they tend to receive more unique names from their parents who are also more likely to be in high economic status. In addition to these points, we can also assume that 3) the participants inherited their parents' economic status. Hence, unique names of the participants may be correlated to their economic status, so I approve the revised draft.

Are arguments sufficiently supported by evidence from the published literature or by new data and results?

Partly

Is the conclusion balanced and justified on the basis of the presented arguments?

Yes

Is the rationale for commenting on the previous publication clearly described?

Yes

Are any opinions stated well-argued, clear and cogent?

Yes

Reviewer Expertise:

social psychology, social survry, culural psycholgy

I confirm that I have read this submission and believe that I have an appropriate level of expertise to confirm that it is of an acceptable scientific standard.

# Reviewer report: Reviewer response for version 1

**DOI:** 10.5256/f1000research.144884.r191660

**Published:** 2023-08-22

**Authors:** Shintaro Fukushima

**Affiliations:** 1 Tokyo Woman's Christian University, Tokyo, Japan

The claims of the author (Dr. Ogihara) are quite reasonable and convince me in general.

 However, the concept of uniqueness and some insistences are confusing or inadequately supported by the references.

 The author insists on a selection bias on a sampled elderly; living elderly at the time when the national survey was conducted would be wealthier (and healthier) compared with the elderly who had already passed away, which results in more uniqueness of the names of the sampled living elderly.

 Although the fact that this systematic selection bias is stronger in the elderly is critical for the intergenerational comparison, the three variables A) unique names of the participants, B) uniqueness of the participants, and C) uniqueness of the parents of the participants seem to be used confusingly in the claims by the author.

 Can A) unique names of the participants be identified as B) psychological uniqueness of the participants? The author says that previous research has demonstrated that wealthy people tend to express more uniqueness, but does it really mean that wealthy people tend to have more unique names? As for the causal direction, money could get people to have freedom and uniqueness as demonstrated in Ma et al. (2017) which is cited in the present paper, but could unique-name also get people to be wealthy? In fact, some studies indicate that unique names limit future employment or social prospects (e.g., Kalist & Lee, 2009; Rogers-Anderson, 2017; Savage & Wells, 1948). In addition, the negative consequence of having unique names might relatively be seen in the interdependent countries such as China where people are expected to have social harmony or rejection avoidance rather than in the independent countries such as the U.S. where people are expected to have independence or uniqueness.

 Furthermore, A) unique names of the participants would be directly affected by C) uniqueness of the parents of the participants rather than by B) uniqueness of the participants themselves. If so, the selection bias the author pointed out might not be as critical as expected.

 Overall, I recommend the author to add empirical evidence which shows that having unique names results in low life expectancy directly or indirectly. In that case, individual-level (within culture) associations rather than social/group-level (between-culture) associations are preferable to be shown because between-culture association includes the mixed effects of A) unique names of the participants, B) uniqueness of the participants, and C) uniqueness of the parents of the participants, which would fail to explain the selection bias of the sampled individuals within China.

Are arguments sufficiently supported by evidence from the published literature or by new data and results?

Partly

Is the conclusion balanced and justified on the basis of the presented arguments?

Yes

Is the rationale for commenting on the previous publication clearly described?

Yes

Are any opinions stated well-argued, clear and cogent?

Yes

Reviewer Expertise:

social psychology, social survry, culural psycholgy

I confirm that I have read this submission and believe that I have an appropriate level of expertise to confirm that it is of an acceptable scientific standard, however I have significant reservations, as outlined above.

## References

[1] : First Names and Crime: Does Unpopularity Spell Trouble?. *Social Science Quarterly* .2009;90(1) : 10.1111/j.1540-6237.2009.00601.x 39-49 10.1111/j.1540-6237.2009.00601.x

[2] : The little book of BOGAN baby names. *New Holland Publishers* .

[3] : A note on singularity in given names. *J Soc Psychol* .1948;27(2) : 10.1080/00224545.1948.9918930 271 10.1080/00224545.1948.9918930 18874626

# Response

**Published:** 2023-11-02

**Authors:** Yuji Ogihara

**Affiliations:** Psychology, Aoyama Gakuin Daigaku, Shibuya, Tokyo, Japan

November 2nd, 2023

 Dear Dr. Shintaro Fukushima,

 Thank you very much for reviewing my manuscript and providing a valuable comment.

 I have modified the manuscript according to your comment. I offer my response to the comment below.

 I have copied and pasted your comment without making changes.

*The claims of the author (Dr. Ogihara) are quite reasonable and convince me in general.*

*However, the concept of uniqueness and some insistences are confusing or inadequately supported by the references.*

*The author insists on a selection bias on a sampled elderly; living elderly at the time when the national survey was conducted would be wealthier (and healthier) compared with the elderly who had already passed away, which results in more uniqueness of the names of the sampled living elderly.*

*Although the fact that this systematic selection bias is stronger in the elderly is critical for the intergenerational comparison, the three variables A) unique names of the participants, B) uniqueness of the participants, and C) uniqueness of the parents of the participants seem to be used confusingly in the claims by the author.*

*Can A) unique names of the participants be identified as B) psychological uniqueness of the participants? The author says that previous research has demonstrated that wealthy people tend to express more uniqueness, but does it really mean that wealthy people tend to have more unique names? As for the causal direction, money could get people to have freedom and uniqueness as demonstrated in Ma et al. (2017) which is cited in the present paper, but could unique-name also get people to be wealthy? In fact, some studies indicate that unique names limit future employment or social prospects (e.g., Kalist & Lee, 2009; Rogers-Anderson, 2017; Savage & Wells, 1948). In addition, the negative consequence of having unique names might relatively be seen in the interdependent countries such as China where people are expected to have social harmony or rejection avoidance rather than in the independent countries such as the U.S. where people are expected to have independence or uniqueness.*

*Furthermore, A) unique names of the participants would be directly affected by C) uniqueness of the parents of the participants rather than by B) uniqueness of the participants themselves. If so, the selection bias the author pointed out might not be as critical as expected.*

*Overall, I recommend the author to add empirical evidence which shows that having unique names results in low life expectancy directly or indirectly. In that case, individual-level (within culture) associations rather than social/group-level (between-culture) associations are preferable to be shown because between-culture association includes the mixed effects of A) unique names of the participants, B) uniqueness of the participants, and C) uniqueness of the parents of the participants, which would fail to explain the selection bias of the sampled individuals within China.*

 Thank you for your important comment. My explanation in the previous version of the article was insufficient. Thus, I have added an explanation as below.

 “Previous research has demonstrated that people of high economic status tend to express more uniqueness (e.g., Ma et al., 2017; Snibbe & Markus, 2005; Stephens et al., 2007; Wang et al., 2020). This leads that they tend to receive more unique names from their parents who are also more likely to be in high economic status. Thus, the values of the uniqueness indicators in the older birth cohorts would be higher than the actual values and should be lower in reality.”

 I provide one possible interpretation of the results, as I wrote in the previous version of the article. The important point is that all six indicators the authors analyzed consistently showed that the unique names did NOT increase from 1920 to 1969. Thus, I recommend that the authors of the target article examine this possibility.

 Thank you for your further consideration of this manuscript.

 I look forward to hearing from you at your earliest convenience.

 Sincerely,

 Yuji Ogihara, Ph.D.

 Department of Psychology, College of Education, Psychology and Human Studies, Aoyama Gakuin University

 Address: 4-4-25 Shibuya, Shibuya-ku, Tokyo, 150-8366, Japan

 E-mail: yogihara@rs.tus.ac.jp

Web: https://sites.google.com/site/yujiogiharaweb/english

# Reviewer report: Reviewer response for version 1

**DOI:** 10.5256/f1000research.144884.r196699

**Published:** 2023-08-18

**Authors:** Gabriela Fatková

**Affiliations:** 1 Department of Anthopology, University of West Bohemia, Sedlacková, Czech Republic

The article discusses and criticizes a study (Bao et al. 2021) on historical changes in given names in China from 1920 to 2005. The author presents three key points of contention. Firstly, it questions whether the samples for older birth cohorts are truly representative due to potential selection biases. The cross-sectional nature of the data and the unsatisfactorily settled biases stemming from it could lead to inaccuracies in the study's conclusions. Secondly, it challenges the study´s claim of an increase in unique names only after the 1970s, pointing out that in the results presented it did not apply to all indicators. Lastly, the author aptly notes that the interpretation and introduction of broad social context (historical, societal, and policy changes) of any quantitative analysis is needed. The suggestions are reasonable and constructive.

 The author recommends a deeper exploration of societal and policy transformations that might underpin the observed fluctuations. In addition to the last point, the broader reflection on the evolving functions of names within Chinese society would be very useful. Names are not mere symbols but integral to a dynamic social process, shaping identities and forging connections among individuals and groups on a daily basis. Name studies approaching names solely as indicators of single social processes, such as individualization in the case of Bao et al. (2021), is deemed shallow and insufficient. Consequently, the author´s critique proposes comprehensive considerations to enhance the validity and impact of the research, encompassing a nuanced examination of the multifaceted roles names play in the fabric of Chinese society.

Are arguments sufficiently supported by evidence from the published literature or by new data and results?

Yes

Is the conclusion balanced and justified on the basis of the presented arguments?

Yes

Is the rationale for commenting on the previous publication clearly described?

Yes

Are any opinions stated well-argued, clear and cogent?

Yes

Reviewer Expertise:

Anthropology of names and naming, gender, kinship, food, memory

I confirm that I have read this submission and believe that I have an appropriate level of expertise to confirm that it is of an acceptable scientific standard.

## References

[1] : Novel Evidence for the Increasing Prevalence of Unique Names in China: A Reply to Ogihara. *Front Psychol* .2021;12: 10.3389/fpsyg.2021.731244 731244 10.3389/fpsyg.2021.731244 34938229 PMC8685573
